# Perivascular Expression and Potent Vasoconstrictor Effect of Dynorphin A in Cerebral Arteries

**DOI:** 10.1371/journal.pone.0037798

**Published:** 2012-05-25

**Authors:** Éva Ruisanchez, Attila Cselenyák, Rege Sugárka Papp, Tamás Németh, Krisztina Káldi, Péter Sándor, Zoltán Benyó

**Affiliations:** 1 Institute of Human Physiology and Clinical Experimental Research, Semmelweis University, Budapest, Hungary; 2 Neuromorphological and Neuroendocrine Research Laboratory, Department of Anatomy, Histology and Embryology, Semmelweis University and Hungarian Academy of Sciences, Budapest, Hungary; 3 Department of Physiology, Semmelweis University, Budapest, Hungary; Massachusetts General Hospital/Harvard Medical School, United States of America

## Abstract

**Background:**

Numerous literary data indicate that dynorphin A (DYN-A) has a significant impact on cerebral circulation, especially under pathophysiological conditions, but its potential direct influence on the tone of cerebral vessels is obscure. The aim of the present study was threefold: 1) to clarify if DYN-A is present in cerebral vessels, 2) to determine if it exerts any direct effect on cerebrovascular tone, and if so, 3) to analyze the role of κ-opiate receptors in mediating the effect.

**Methodology/Principal Findings:**

Immunohistochemical analysis revealed the expression of DYN-A in perivascular nerves of rat pial arteries as well as in both rat and human intraparenchymal vessels of the cerebral cortex. In isolated rat basilar and middle cerebral arteries (BAs and MCAs) DYN-A (1–13) and DYN-A (1–17) but not DYN-A (1–8) or dynorphin B (DYN-B) induced strong vasoconstriction in micromolar concentrations. The maximal effects, compared to a reference contraction induced by 124 mM K^+^, were 115±6% and 104±10% in BAs and 113±3% and 125±9% in MCAs for 10 µM of DYN-A (1–13) and DYN-A (1–17), respectively. The vasoconstrictor effects of DYN-A (1–13) could be inhibited but not abolished by both the κ-opiate receptor antagonist *nor*-Binaltorphimine dihydrochloride (NORBI) and blockade of G_i/o_-protein mediated signaling by pertussis toxin. Finally, des-Tyr^1^ DYN-A (2–13), which reportedly fails to activate κ-opiate receptors, induced vasoconstriction of 45±11% in BAs and 50±5% in MCAs at 10 µM, which effects were resistant to NORBI.

**Conclusion/Significance:**

DYN-A is present in rat and human cerebral perivascular nerves and induces sustained contraction of rat cerebral arteries. This vasoconstrictor effect is only partly mediated by κ-opiate receptors and heterotrimeric G_i/o_-proteins. To our knowledge our present findings are the first to indicate that DYN-A has a direct cerebral vasoconstrictor effect and that a dynorphin-induced vascular action may be, at least in part, independent of κ-opiate receptors.

## Introduction

Although the perivascular nerves of brain vessels were already recognized by Thomas Willis in the 17^th^ century [1], their role in the regulation of cerebral circulation is still poorly understood [2], [3]. Since the development of the metabolic homeostasis concept by Roy and Sherrington in 1890 [4], cerebrovascular research has been focused primarily on paracrine mediators of cerebral blood flow (CBF) regulation. However, recent advances in functional neuroimaging techniques have called our attention to vascular reactions which cannot be completely explained by this paradigm [5], [6]. Most notably, it has been clearly shown that changes in CBF precede the release of metabolic and endothelium-derived mediators during neuronal activation. Furthermore, in some disorders of the cerebral circulation, like vascular migraine headache, vasomotion and vasospasm following subarachnoid hemorrhage or traumatic brain injury, CBF is completely uncoupled from the metabolic demands of neurons. Therefore, the concept of neural regulation and the “functional neurovascular unit” has been recently reappraised [2], [6].

Cerebral vessels receive dense innervation either via the peripheral nervous system or directly from the central nervous system (CNS) [2], [3]. The former is often called “extrinsic innervation” and includes sympathetic, parasympathetic and sensory nerve fibers. In contrast, “intrinsic innervation” of the cerebral vessels includes local interneurons and nerve fibers originating from the locus coeruleus, raphe nuclei, basal forebrain, thalamus and nucleus fastigii of the cerebellum. The most important neurotransmitters of cerebral perivascular nerves are norepinephrine, acetylcholine, nitric oxide, serotonin, glutamate, γ-aminobutyric acid and various neuropeptides including opioid peptides [7], [8]. Enkephalins have been identified in cerebral perivascular nerve fibers of humans, pigs, cats, rabbits, pigeons and rats [9]–[13]. In swine middle cerebral arteries (MCAs) Met- and Leu-enkephalin immunoreactivity was localized in large dense core vesicles of noradrenergic perivascular nerves [10], [11]. Interestingly, sympathoadrenal stimulation by acute cerebral ischemia (Cushing response) induced a 70% decrease of the MCA enkephalin content, an effect that was absent after the depletion of catecholamines, indicating an interaction between the sympathetic and enkephalinergic regulation of the cerebral circulation.

Beside of enkephalins other endogenous opioid peptides, like β-endorphin and dynorphins, have also been implicated in cerebrovascular regulation, but their expression in the cerebral vasculature is less documented [8], [14]. In 1986 Moskowitz and colleagues described the presence of dynorphin B (DYN-B) in guinea-pig cerebral perivascular nerves [15], an observation that has been confirmed subsequently by the same authors in rats [16] and later by others in monkeys [17]. Furthermore, measurable levels of DYN-B were detected in isolated human, canine, bovine and feline cerebral vessels [16]. However, the potential role of DYN-B in the regulation of cerebral circulation remained obscure, since it failed to induce any effect in cerebral arteries isolated from dogs [15] or rats [17]. In the present study we aimed to investigate the cerebrovascular expression and potential vasoactive effects of the other member of the dynorphin family, dynorphin A (DYN-A). Functional experiments were performed in isolated basilar arteries (BAs) and MCAs, representing two different regions of pial vessels, because with this approach the direct vascular effects of DYN-A could be determined. Since we found that DYN-A is present in perivascular nerves of cerebral arteries and induces sustained vasoconstriction, we further investigated the roles of κ-opiate receptors and G_i/o_ proteins in mediating this vasoactive effect.

## Methods

### Ethics Statement

The experiments were performed according to the guidelines of the Hungarian Law of Animal Protection (243/1988), and all procedures were approved by the Semmelweis University Committee on the Ethical Use of Experimental Animals (590/99 Rh).

### Immonohistochemistry

Adult male Wistar rats (350–450 g bw.) were anesthetized with 80 mg/kg Ketamine (CP Pharma, Burgdorf, Germany) and 160 mg/kg Primazin (Alfason, Woerden, Netherlands) and perfused transcardially with 0.9% saline followed by 300 ml of 4% paraformaldehyde, pH 7.4. The brains were removed and post-fixed overnight in a fresh solution at 4°C.

Pial vessels (BA, MCAs and their main branches) were immunostained as whole-mount preparations. After washing in 0.1 M phosphate buffer (PB) pH 7.4, the specimens were treated with 0.5% Triton X-100 and 10% normal goat serum for 1 h each at room temperature. The endogenous peroxidase activity was blocked by a 15-min incubation in 3% H_2_O_2_ (Reanal, Budapest, Hungary). In order to visualize perivascular nerve fibers the vessels were incubated in mouse anti-synaptophysin antibody (1∶1000; Millipore, Budapest, Hungary) for 36 h at 4°C. Alexa Fluor 633 labeled anti-mouse secondary antibody (1∶500, made in goat, Invitrogen, Carlsbad, CA, USA) was used for 1 h at room temperature. Immunohistochemistry of DYN-A was performed as described below for brain sections.

Expression analysis of cortical intraparenchymal vessels were performed in 50 µm thick horizontal sections of rat brains prepared and post-fixed as described above, and cryoprotected with 20% sucrose for 24 h at 4°C. Small blocks of human brain (from Human Brain Tissue Bank, Semmelweis University, Budapest) were cut from the temporal cortex (post mortem delay: 10 h), fixed in 4% paraformaldehyde for 48 h at pH 7.4, and cryoprotected with 20% sucrose for 24 h at 4°C. Serial, 50 µm thick coronal sections were cut on a frigomobile (Frigomobil, Reichert-Jung, Vienna, Austria).

After washing in 0.1 M PB pH 7.4, sections were treated with 0.5% Triton X-100 and 10% normal goat serum for 1 h each at room temperature. The endogenous peroxidase activity was blocked by a 15-min incubation in 3% H_2_O_2_ (Reanal, Budapest, Hungary). Sections were next incubated in anti-DYN-A (1–13) antibody (1∶2500, made in rabbit, Peninsula Laboratories Inc., San Carlos, CA, USA) for 36 h at 4°C, in biotinylated anti-rabbit secondary antibody (1∶1000, made in goat, Vector Laboratories, Burlingame, CA, USA) for 1 h at room temperature and in avidin–biotin–horseradish peroxidase complex (1∶500, Vectastain ABC Elite kit, Vector Laboratories, Burlingame, CA, USA) for 1 h at room temperature. The sections were then incubated in FITC-tyramide (1∶10,000) and 0.015% H_2_O_2_ in 0.05 M Tris buffer at pH 8.2 for 10 min. Between the incubation steps, the sections were rinsed three times for 10 min in PB. Western blot analysis of the specificity of the primary antibody revealed similar affinities to DYN-A (1–13) and DYN-A (1–17), but no cross-reaction with DYN-A (1–8) or DYN-B.

Sections were mounted on Superfrost slides, and the slices were coverslipped with Aqua-Poli Mount (Polysciences, Inc., Warrington, USA). Confocal images of slides and whole mount preparations were acquired with a Zeiss LSM 510 META (Carl Zeiss, Jena, Germany) confocal laser scanning microscope using a 20X DIC (Plan Apochromat, NA = 0.80) or 63X DIC (Plan Apochromat oil immersion, NA = 1.4) objective and processed by LSM AIM 4.0 software.

### Myograph experiments

Adult male Wistar rats (350–450 g bw.) were exsanguinated rapidly under deep ether anesthesia and 2–3 mm long ring segments of the BAs and MCAs were prepared as described previously [18], [19]. Special care was taken to preserve the endothelium during preparation. The segments were transferred to 6-mL organ baths of a conventional myograph system (610 M, Danish Myo Technology A/S, Aarhus, Denmark) filled with a modified Krebs solution of the following composition (mmol/L): NaCl 119, KCl 4.7, KH_2_PO_4_ 1.2, CaCl_2_·2H_2_O 2.5, MgSO_4_·7H_2_O 1.2, NaHCO_3_ 20, EDTA 0.03 and glucose 10. The bath solution was bubbled continuously with carbogen (95% O_2_/5% CO_2_) in order to maintain a pH of 7.4. The vessel segments were mounted on two L-shaped tungsten wires (75 and 50 µm in diameter for BA and MCA, respectively): one wire was fixed to the wall of the bath, and the other was fixed to a force transducer. Isometric vascular tensions were recorded by the MP100 data acquisition system and analyzed with the AcqKnowledge 3.8.2 software of BIOPAC Systems (Goleta, CA, USA).

The vessels were allowed a 45-minute equilibration period, during which the resting tension was adjusted to 3.5–4.5 mN (BA) or 1.5–2.5 mN (MCA), and the bath solution was warmed to 37°C with repeated washing in every 15 minutes. Thereafter, each segment was exposed to 124 mmol/L K^+^ Krebs solution to elicit a reference contraction. After a 30-min resting period, the effects of dynorphins were tested either on the resting tension or after precontraction with 10 µM serotonin (BA) or 100 µM UTP (MCA). (Since the results of the first experiments clearly showed that none of the tested dynorphins induce any relaxation in a precontracted vessel, in all further experiments they were applied on the resting tension in order to study their contractile effects.) At the end of the experiments, the functional integrity of the endothelium was tested by cumulative application of 0.01 to 10 µM bradykinin (MCA) or acetylcholine (BA) after induction of precontraction as described above. According to results of previous studies [18], [20] segments that did not exhibit at least 40% (BA) or 20% (MCA) relaxation of the precontraction were considered to have damaged endothelium and were excluded from the study.

Some segments were pretreated with pertussis toxin (PTX) before mounting on the myograph in order to evaluate the role of heterotrimeric G_i/o_-proteins in mediating the vascular effects of DYN-A. These vessels were incubated with 3 µg/ml PTX overnight at 37°C and vehicle-treated vessels undergoing the same procedure served as controls.

Changes in vascular tension are expressed as percentage of the reference contraction induced by 124 mmol/L K^+^ Krebs solution and presented as mean±SEM. Statistical analysis was performed using one-way ANOVA followed by Tukey's post hoc test or Student's t-test, as appropriate; n represents the number of experiments. A P value of less than 0.05 was considered to be statistically significant.

DYN-A (1–13) was from Merck KGaA (Darmstadt, Germany), DYN-A (2–13) was synthesized by CASLO Laboratory ApS (Lyngby, Denmark). Other dynorphins used in this study were from Bachem AG (Bubendorf, Switzerland). Dynorphins were freshly dissolved on the day of the experiment and were kept on ice until administration. PTX, bradykinin and *nor*-Binaltorphimine dihydrochloride (NORBI) were from Tocris Bioscience (Bristol, UK). All other chemicals were obtained from Sigma-Aldrich (St. Louis, MO, USA).

## Results

**Figure 1:**
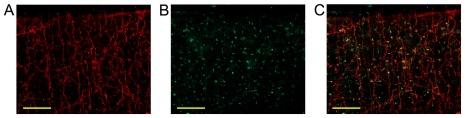
Dynorphin A (DYN-A) is present in perivascular nerves of the rat basilar artery. Localization of DYN-A in perivascular nerve fibers of rat basilar artery by immunofluorescent confocal microscopy. Anti-synaptophysin staining (red fluorescence) indicates the synaptic vesicles of perivascular nerves on the surface of the basilar artery (A). DYN-A (1–13) (green fluorescence) is abundantly found in the adventitia (B) and co-localized with synaptophysin (C). Bar = 100 µm.

**Figure 2:**
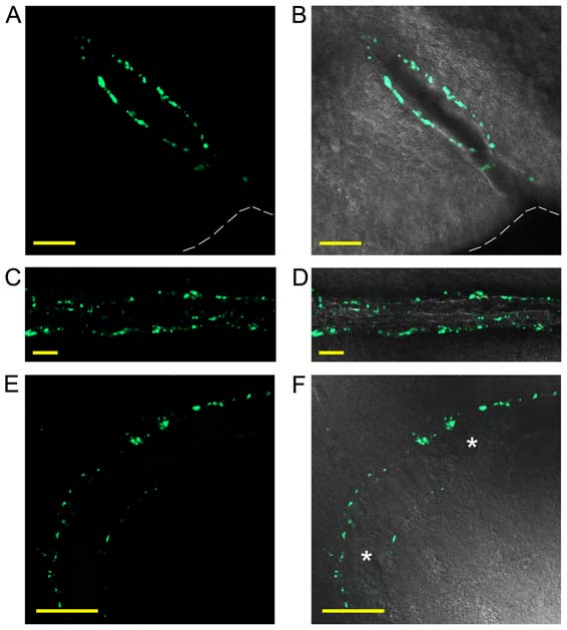
Dynorphin A (DYN-A) is present in perivascular nerves of rat and human intraparenchymal arteries. Oblique sections of rat (A and B) as well as longitudinal sections of rat (C and D) and human (E and F) cerebral arteries are shown. Merged images of DYN-A (1–13) immunoreactivity (green fluorescence) and transmitted-light (grayscale) indicate the perivascular localization of DYN-A. Broken line shows the surface of the brain on panels A and B, asterisks indicate red blood cell clots in the lumen of the artery on panel F. Bar = 50 µm on each panel.

**Figure 3:**
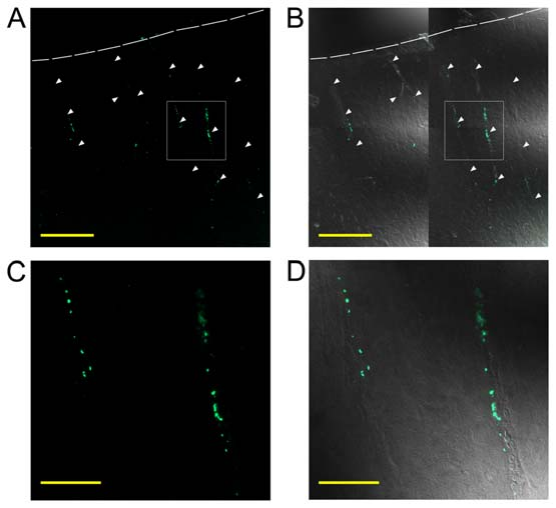
Dynorphin A (DYN-A) is present in perivascular nerves of the rat intraparenchymal arterioles. Several arterioles (indicated by arrows) are present in tile scan images (A and B) of a 900×900 µm large cortical area. C and D panels show the selected region of the upper panels. Overlay of DYN-A (1–13) immunoreactivity (green fluorescence) and transmitted light images (grayscale) on panels B and D indicate the perivascular expression of DYN-A. Broken line shows the surface of the brain. Bar indicates 200 µm on upper panels and 50 µm on lower panels.

Rat pial arteries, including the BA and MCA, ubiquitously showed DYN-A immunoreactivity in the adventitia of the vessels. DYN-A was co-localized with synaptophysin, indicating its presence in presynaptic vesicles (Figure 1). DYN-A immunoreactive perivascular nerves followed the vessels after their entry into the brain parenchyma (Figure 2A and 2B) and surrounded approximately 50% of the intraparenchymal arteries of the cerebral cortex (Figure 2C and 2D). Three-dimensional reconstruction of brain slices clearly showed the network of DYN-A positive nerves around the vessels (Supplementary Video S1). Interestingly, human cerebrocortical arteries showed a similar pattern of DYN-A expression (Figure 2E and 2F). Furthermore, dynorphinergic nerves were also observed around rat cerebrocortical arterioles (Figure 3), which play a major role in the regulation of the local tissue blood flow. In longitudinal sections of intraparenchymal arterioles it was obvious that the density of DYN-A positive fibers varies significantly from segment to segment of the vessel (Figure 3A and 3B) but none of the cerebrocortical layers appeared to show higher or lower perivascular DYN-A expression.

**Figure 4:**
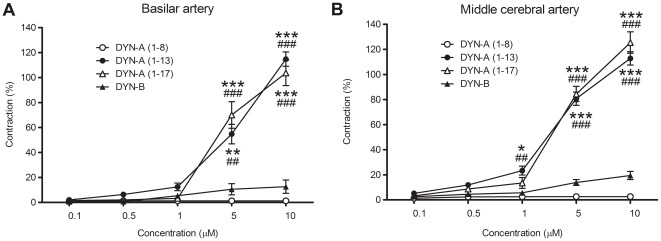
Dynorphin A (DYN-A) (1–13) and (1–17) induce strong contraction of rat cerebral arteries. Effects of cumulative concentrations of DYN-A (1–8), (1–13), (1–17) and Dynorphin B (DYN-B) on the resting tension of rat basilar (A) and middle cerebral (B) arteries. DYN-A (1–13) and DYN-A (1–17) induce strong, dose-dependent vasoconstriction in both vessels, whereas DYN-A (1–8) and DYN-B have no significant effect. Values are expressed as mean±SEM percentage of the reference contraction induced by 124 mmol/L K^+^ Krebs solution, n = 6–37. *P<0.05, **P<0.01, ***P<0.001 vs. DYN-A (1–8); ^##^ *P*<0.01, ^###^ *P*<0.001 vs. DYN-B.

The potential vasoactive effects of naturally occurring dynorphins [DYN-A (1–8), (1–13), (1–17) and DYN-B] were determined both on the resting tension and after precontraction of rat cerebral arteries. No relaxant effect of any of the tested peptides was observed, although the functional integrity of the endothelium and the ability of the vessels to relax were verified by the normal reactivity of the BAs and MCAs to acetylcholine and bradykinin, respectively (data not shown). In contrast, when applied on the resting tension, both DYN-A (1–13) and DYN-A (1–17) but not DYN-A (1–8) induced very strong vasoconstriction in micromolar concentrations (Figure 4). The vasoconstrictor effects of 10 µM DYN-A (1–13) and DYN-A (1–17) were stronger than the reference contraction induced by 124 mM K^+^, and did not decline within 10 minutes until the baths were washed with fresh Krebs solution. In contrast, the effect of DYN-B was negligible (Figure 4).

**Figure 5:**
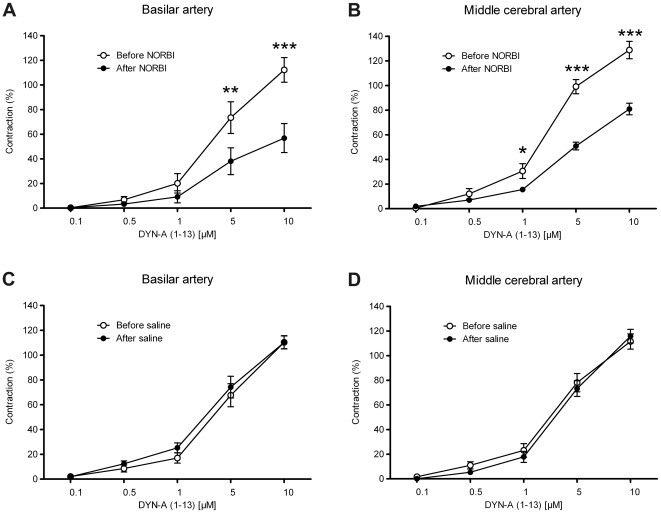
Dynorphin A (DYN-A) induced cerebral vasoconstriction is partly mediated by κ-opiate receptors. Effect of the κ-opiate receptor antagonist *nor*-Binaltorphimine dihydrochloride (NORBI, 50 µM) on the responsiveness of rat basilar (A) and middle cerebral (B) arteries to DYN-A (1–13). NORBI was able to reduce the vasoconstrictor effect of DYN-A in both vessels, whereas its vehicle (saline) was without any effect (C and D). Values are expressed as mean±SEM percentage of the reference contraction induced by 124 mmol/L K^+^ Krebs solution, n = 8–10. Asterisks indicate significant (**P<0.01, ***P<0.001) differences between values before and after NORBI treatment.

**Figure 6:**
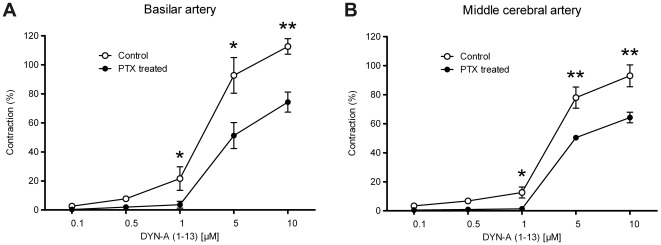
Dynorphin A (DYN-A) induced cerebral vasoconstriction is partly mediated by heterotrimeric G_i/o_-proteins. Effect of the inhibition of G_i/o_-signaling with pertussis toxin (PTX, applied as described in “Methods”) on the responsiveness of rat basilar (A) and middle cerebral (B) arteries to DYN-A (1–13). In control vessels the slightly weaker reactions to DYN-A (as compared to the previous figures) were probably the consequence of overnight incubation in the Krebs solution containing the vehicle of PTX. PTX inhibited the vasoconstrictor effects of DYN-A. Values are expressed as mean±SEM percentage of the reference contraction induced by 124 mmol/L K^+^ Krebs solution, n = 6–8. Asterisks indicate significant (*P<0.05, **P<0.01) differences between PTX-treated and vehicle-treated control vessels.

**Figure 7:**
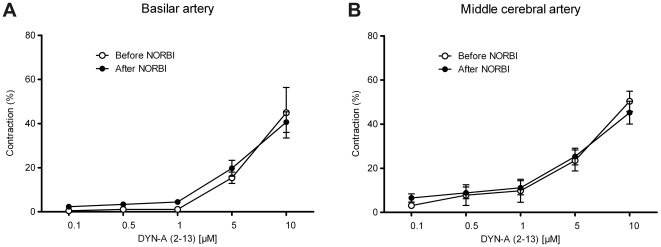
Dynorphin A (DYN-A) (2–13) induces contraction of rat cerebral arteries independently of κ-opiate receptors. Effects of DYN-A (2–13) on the tone of rat basilar (A) and middle cerebral (B) arteries before and after treatment with the κ-opiate receptor antagonist *nor*-Binaltorphimine dihydrochloride (NORBI, 50 µM). DYN-A (2–13), which is reportedly inactive at opiate receptors, evoked weaker [in comparison to DYN-A (1–13) shown in previous figures] but significant contractions in both vessels, and these effects were resistant to NORBI. Values are expressed as mean±SEM percentage of the reference contraction induced by 124 mmol/L K^+^ Krebs solution, n = 6–9.

The κ-opiate receptor antagonist NORBI was able to reduce the vasoconstrictor effect of DYN-A (1–13) both in BAs (Figure 5A) and MCAs (Figure 5B), whereas its vehicle (saline) was without any effect (Figures 5C and 5D). Furthermore, inhibition of G_i/o_-mediated signaling with PTX also inhibited the vasoconstrictor effects of DYN-A (1–13) (Figures 6A and 6B), indicating the involvement of G_i/o_-coupled κ-opiate receptors in the mediation of the effect. However, neither NORBI nor PTX was able to abolish the DYN-A-induced cerebrovascular constriction completely, indicating that a κ-opiate receptor-independent mechanism may also contribute to its mediation. In accordance with this assumption, DYN-A (2–13), which is inactive at opiate receptors, was also able to induce weaker but significant vasoconstriction in both BAs (Figure 7A) and MCAs (Figure 7B), and these effects were resistant to NORBI (Figure 7A and 7B).

## Discussion

Various and in many cases conflicting data have been published on the roles of opioid peptides, opiates and their receptors in cerebral circulation. In a review published in 1996, based on the literature available at the time, we drew the conclusion that the endogenous opioid system represents a latent regulatory mechanism, which is of limited importance under resting conditions but may significantly influence cerebral circulation in pathophysiological states [8]. Although numerous papers have been published on this field in the last 16 years as well, the effects of endogenous opiates on cerebrovascular functions are still obscure. In the present study we show that DYN-A is present in cerebral perivascular nerves and induces strong vasoconstriction, which is mediated by both κ-opiate receptor-dependent and -independent mechanisms.

There are numerous controversies in the literature regarding the role of dynorphins and κ-opiate receptors in cerebral circulation [8]. In the early studies of Altura et al. some synthetic agonists of κ-opiate receptors induced vasorelaxation, whereas others brought about constriction in canine cerebral arteries [21], [22]. However, the mediation of the vasoconstrictor effect was attributed to σ-receptors. In 1986 Sándor et al. demonstrated that intracerebroventricular (ICV) administration of DYN-A (1–13) results in a reduction of the hypothalamic blood flow without changing global CBF or cerebral blood volume in rats [3]. However, the question whether DYN-A had a direct vasoconstrictor effect, or the reduction of the blood flow was secondary to changes in neurovascular regulation and/or neuronal metabolic activity remained open.

The role of dynorphins in cerebrovascular regulation has been studied extensively by Armstead and co-workers in newborn and juvenile piglets and led to the following main findings. DYN-A (1–13) induced vasodilation in pial arteries and arterioles, which was reversed to vasoconstriction during hemorrhagic hypotension or sustained hypoxia as well as after fluid percussion brain injury [23]–[29]. Since DYN-A failed to influence the cerebral metabolic rate of glucose [26], an indirect effect on pial vessels through modulation of the neuronal metabolic activity could be excluded. It was also shown that the vasodilator effect of DYN-A is mediated by NO and probably by the activation of ATP- and Ca^++^-sensitive K^+^-channels [30]–[32]. Interestingly, DYN-A increased the vasopressin concentration in the cerebrospinal fluid, an effect that appeared to be responsible for the pial arterial vasoconstriction induced by DYN-A during hemorrhagic hypotension and sustained hypoxia as well as after traumatic brain injury [27], [29], [33], [34].

In our present study none of the naturally occurring dynorphins induced any vasorelaxant effect in isolated cerebral vessels of adult rats, in spite of the facts that rat cerebrovascular endothelial cells do express κ-opiate receptors [35], and the functional integrity of the endothelium has been verified in our experiments. Although this discrepancy between our results and those of Armstead et al. could be explained as age- or species-related differences in cerebrovascular reactivity, we suggest that it is due to the different experimental approaches. Since in newborn pigs DYN-A-induced pial vasorelaxation was accompanied by an almost three-fold increase in cyclic GMP concentration in the cerebrospinal fluid [30], it is likely that it was mediated by neuronal release of NO. In contrast, in our *in vitro* experiments only the direct vascular effects of DYN-A were observed. However, the cellular mechanism of the dynorphin action cannot be identified exactly from our results either. It is unlikely that perivascular nerves would mediate the vasoconstriction, since even if they expressed κ-opiate receptors one would expect a decrease of neurotransmitter release upon their activation by DYN-A. On the other hand, the endothelium may be involved in the vasoconstrictor effect of DYN-A, since it express κ-opiate receptors [35], which were shown to mediate endothelin-release [36]. Finally, DYN-A may directly stimulate smooth muscle contraction as it has been demonstrated in the gastrointestinal system [36]–[41].

Endogenous opioid peptides have been implicated as pathophysiological factors in the development of traumatic and ischemic lesions of the CNS [42]. Elevated DYN-A levels together with upregulation of κ-opiate receptors were reported at regions of spinal cord injury [43]–[45], and κ-opiate receptor antagonists were able to prevent posttraumatic neurological deficits [46], [47]. Furthermore, it has been demonstrated that (i) intrathecal infusion of DYN-A by itself is able to induce energy depletion, edema formation and neurologic motor dysfunction in the spinal cord [48]–[51], and (ii) both ICV and systemic administration of DYN-A and synthetic κ-opiate agonists prior to induction of brain injury worsens the posttraumatic neurological outcome [52]. In our experiments vertebral arteries, similarly to BAs and MCAs, also developed contraction in response to DYN-A (data not shown), an observation that may elucidate the mechanism of dynorphin-related traumatic spinal cord injury. Consistently with this hypothesis, DYN-A (1–8), which was without any vasoconstrictor effect in our present study, failed to reproduce the detrimental consequences of intrathecal infusion of DYN-A (1–13) or DYN-A (1–17) [48]–[50].

An important finding of the present study is that the cerebrovascular effect of DYN-A is only partly mediated by κ-opiate receptors, and DYN-A (2–13), which lacks the amino terminal tyrosine and therefore the affinity to opiate receptors, can also induce cerebral vasoconstriction. Since aminopeptidase-mediated conversion is a common route of dynorphin-metabolism, significant amounts of DYN-A (2–13) and DYN-A (2–17) may be present *in vivo* [52], [53]. For instance, DYN-A (2–13) was reported to be one of the main DYN-A (1–13) metabolite both in the human plasma and cerebrospinal fluid [54], [55]. Therefore, the cerebral vasoconstriction induced by DYN-A (1–13) may persist after the degradation of the peptide.

It has been shown in neuronal cells and membrane preparations that DYN-A may bind to several non-opioid receptors including muscarinic receptors [56], N-methyl-D-aspartate (NMDA) receptors [57], [58], the orphanin receptor [59], melanocortin receptors [60], neuropeptide Y and peptide YY receptors [61], the membrane glucocorticoid receptor [62] and bradykinin-receptors [63]. Further studies will be required to clarify whether it is any of these receptors or other mechanisms that mediate the non-κ-opiate receptor related vasoconstrictor effect of DYN-A. It is noteworthy, however, that DYN-A induced CNS injury is also partly independent of κ-opiate receptors [64]–[66], a similarity that underlines the potential role of the vasoconstrictor effect of DYN-A in the development of the posttraumatic neurological dysfunction.

In conclusion, the present study shows that DYN-A (1–13) and/or DYN-A (1–17) are present in cerebral perivascular nerves and both are able to induce sustained contraction of cerebral arteries, whereas DYN-A (1–8) and DYN-B have no such effect. The DYN-A-induced vasoconstriction is only partly mediated by κ-opiate receptors and heterotrimeric G_i/o_-proteins. To our knowledge, our present findings are the first to indicate that a dynorphin-induced vascular effect may be, at least in part, independent of κ-opiate receptors.

## Supporting Information

Video S1 **Perivascular localization of Dynorphin A (DYN-A) in a rat cerebral artery.** Three-dimensional reconstruction of a 50 µm thick brain slice containing an oblique section of a cerebral artery. Purple color indicates the autofluorescence of the arterial media layer, whereas green fluorescence corresponds to DYN-A immunostaining. DYN-A positive nerve fibers are shown to closely entwine the vascular smooth muscle.
(AVI)
